# Myeloid neoplasms after CD19-directed CAR T cells therapy in long-term B-cell lymphoma responders, a rising risk over time?

**DOI:** 10.1038/s41375-025-02605-7

**Published:** 2025-04-24

**Authors:** Nicolas Gazeau, David Beauvais, Rémi Tilmont, Micha Srour, Emmanuelle Ferrant, Violaine Safar, Ludovic Fouillet, Pascale Flandrin-Gresta, Nicolas Gower, Paul Chauvet, Nicolas Duployez, Benjamin Podvin, Julie Demaret, Sarah Huet, Pierre Sujobert, Hervé Ghesquières, Gandhi Damaj, Emmanuel Bachy, Franck Morschhauser, Ibrahim Yakoub-Agha, Maël Heiblig, Pierre Sesques

**Affiliations:** 1 Hematology Department, Centre Hospitalier Universitaire de Lille, Lille, France; 2 Hematology Department, Hospices Civils de Lyon, Pierre Bénite, Lyon, France; 3 Hematology Department, Centre Hospitalier Universitaire de Saint-Etienne, Saint-Etienne, France; 4 Laboratory of Hematology and molecular biology, Hopital Nord, Saint-Etienne, France; 5 CHU de Lille, Université de Lille, Inserm UMR1277, CNRS UMR9020-CANTHER, Lille, France; 6 Biology and Pathology Center, Laboratory of Hematology, Centre Hospitalier Universitaire de Lille, Lille, France; 7 Biology and Pathology Center, Laboratory of Immunology, Centre Hospitalier Universitaire de Lille, Lille, France; 8 Laboratory of Hematology, Centre Hospitalier Universitaire Lyon-Sud, Hospices Civils de Lyon, Pierre-Benite, France; 9 Hematology Department, Centre Hospitalier Universitaire de Caen, Caen, France; 10 CHU de Lille, Université de Lille, INSERM U1286, Infinite, 59000 Lille, France

**Keywords:** Lymphoma, Myelodysplastic syndrome

## Abstract

Therapy-related myeloid neoplasms (t-MN), including myelodysplastic neoplasms (t-MDS) and acute myeloid leukemia (t-AML), have emerged as significant late complications after CAR T cell therapy. We retrospectively analyzed 539 patients with B cell lymphoma treated with CD19 directed CAR T cell therapy across four French centers. Cumulative incidences of t-MN was estimated with relapse or death treated as competing risk. Univariate and propensity score matching (PSM) analyses were conducted to assess risk factors with age and the number of prior treatments as covariates. After a median follow-up of 25 months, the cumulative incidence of t-MN was 4.5% at 2 years. T-MN occurred predominantly as t-MDS (62%) and t-AML (38%) with high cytogenetic risk. Median overall survival after t-MN diagnosis was 4.5 months. In univariate analysis, older age (*p *< 0.01), higher MCV (*p *< 0.01), and higher ICANS grade (*p *= 0.04) were associated with increased risk of t-MN. After PSM, MCV and ICANS grade remained significant risk factors. CAR T cell products with CD28 co-stimulatory domains trended towards higher t-MN risk (*p *= 0.09). NGS analysis showed that 85.7% of t-MN had pre-existing mutations, most commonly TP53. This study highlights t-MN as a severe late complication of CAR T cell therapy. MCV and ICANS grade were identified as key risk factors.

## Introduction

Chimeric antigen receptor T cells (CAR T cells) have revolutionized the treatment of B-cell non Hodgkin lymphoma (B-NHL). Axicabtagene ciloleucel (axi-cel), tisagenlecleucel (tisa-cel), lisocabtagene maraleucel (liso-cel) and brexucabtagene autoleucel (brexu-cel) are four CD19 directed CAR T cells approved by the Food and Drug Administration (FDA) and the European Medicines Agency for the treatment of relapse or refractory (R/R) diffuse large diffuse B cell lymphoma (DLBCL), indolent lymphoma and mantle cell lymphoma (MCL) [1–6]. A significant proportion of patients appear to be long-responders and might be cured after their use [7–11]. Moreover, this treatment is being given earlier for R/R B-NHL, expanding its application to more patients [12–14].

Long-term follow-up of these patients showed that cytopenia appeared to be the most frequent severe and late complication in long-term responding patients [15, 16]. In addition, secondary malignancies, particularly therapy-related myeloid neoplasms (t-MN), have emerged as a growing concern. Patients treated by axi-cel in the ZUMA-1 study showed 3% (*n *= 3) of therapy-related myelodysplastic neoplasms (t-MDS) while the ZUMA-7 study comparing axi-cel to the standard of care (SOC) in second line for B-NHL observed one fatal therapy-related acute myeloid leukemia (t-AML), one t-MDS, one multiple myeloma and no new hematological event in the SOC arm [7, 8]. The TRANSCEND study, using liso-cel, showed 3,7% of t-MN including 8 t-MDS and 2 t-AML while the TRANSFORM study reported 3 second primary malignancies (SPMs) in the liso-cel arm and 7 (including 3 after crossover) in the SOC arm without specifying the number of t-MN [3, 10]. The three years follow up of brexu-cel in the ZUMA-2 study revealed two fatal secondary malignancies [11].

Recently, the FDA Adverse Events Reporting System (FAERS) reported 4.3% of SPMs [17]. While T-cell malignancies, such as T-cell lymphomas were expected to be the main concern as secondary neoplasms following CAR T cell therapy, the most frequently observed malignancies were actually t-AML, and t-MDS [18–21]. Meta-analyses have recently reported that secondary cancers, mainly myeloid neoplasms, are the second leading cause of non-relapse mortality (NRM) behind infection, with rates comparable to those seen with standard therapy [22, 23].

While most studies focused on the frequency of solid and liquid SPMs within trial populations, detailed epidemiological data on the incidence of SPMs remain limited. In this study, we aimed to address this gap by analyzing the incidence and characteristics of t-MN in a heterogenous cohort of patients treated with CD19 directed CAR T cells therapy for R/R B-NHL across four French centers.

## Methods

### Patients

This was a retrospective multicenter study conducted in 4 French centers. All patients were treated with CD19 directed CAR T cells for B-NHL between 2017 and 2023. We first estimated the incidence of t-MN following CD19 directed CAR T cell therapy. Then we identified risk factors associated with their occurrence and evaluated the outcomes of patients who develop t-MN. Patients could be treated under temporary authorization for use (ATU), under post-ATU authorization, under market authorization or in a therapeutic trial (three investigational CAR T cells targeting CD19 included in the study).

A uniform data collection form with embedded data dictionary was provided to all participating centers, along with an example on guidelines for data collection. All sites returned data to the coordinating center. A quality control check was performed by the coordinating center and queries were issued for missing data or data that did not follow the format specified in the data collection form. Datasets are available upon request to the corresponding author.

### Ethics approval and consent to participate

This retrospective study was conducted in accordance with the Declaration of Helsinki. The research protocol complies with the MR003 reference methodology as per the guidelines of the Commission Nationale de l’Informatique et des Libertés (CNIL). All patients who received CAR T-cell therapy provided written informed consent, authorizing the collection and use of their laboratory and clinical data for research purposes related to CAR T-cell therapy.

### Toxicity and treatment evaluation

Response to CAR T cell therapy was defined as the response at one month after infusion using Lugano 2014 criteria for DLBCL.^14^ Overall survival (OS) and progression-free survival (PFS) are described from the time of CAR T-cell infusion unless otherwise specified.

Cytokine release syndrome (CRS) and immune effector cell-associated neurotoxicity syndrome (ICANS) after CAR T cell therapy were graded using chart review according to the ASTCT (American Society of Transplant and Cellular Therapy) consensus grading system [24]. Severe CRS or ICANS was defined as grade 3 or more. NRM was defined as death from any cause occurring without relapse of the lymphoma.

### t-AML and t-MDS analysis

Cytogenetics including standard metaphase karyotyping and fluorescent in situ hybridization were performed locally in each center. Cytogenetic risk was stratified by current European LeukemiaNet (ELN) guidelines [25]. MDS patients’ stratification was based on International Prognostic Scoring System (IPSS), Revised IPSS (IPSS-R) and Molecular IPSS (IPSS-M) [26–28]. Gene mutation analysis was performed by next-generation sequencing (NGS) using a panel of gene depending on the center (supplementary Table S1). Samples were available for some patients who developed t-MN prior to CAR T cell infusion, and retrospective NGS was therefore performed on blood, bone marrow, or the apheresis product for a total of 14 out of the 29 patients who developed t-MN.

### Statistical analysis

Categorical variables were expressed as numbers (percentage) and continuous variables as medians (interquartile range). Cumulative incidences of t-MN and NRM were estimated accounting for relapse or death as competing events. Overall survival of the 29 t-MN patients, censored at the study end-point, was estimated using Kaplan-Meier method from t-MN diagnostics.

A propensity score was estimated using a non-parsimonious multivariable logistic regression model with occurrence of t-MN as outcome and age and the number of prior treatment lines (≤3 or ≥4) before CAR T cell therapy as covariates. Propensity score matching (PSM) was then applied, in a 1:2 ratio, using nearest neighbor method. To evaluate the bias reduction after using PSM, absolute standardized differences (ASD) in prespecified confounders were calculated before and after PSM; an ASD > 10% was interpreted as a meaningful difference. The effects of covariates on the occurrence of t-MN were assessed using the Fine and Gray model before and after PSM. The covariates, mean corpuscular volume (MCV), LDH, absolute lymphocyte count (ALC), CAR T cell product, CRS grade, ICANS grade, and CAR-HEMATOTOX score were chosen with MCV, LDH, ALC, CRS or ICANS grade and CAR-HEMATOTOX score treated as continuous variables [29]. To compare CAR T cell products, treatments were grouped by their co-stimulatory domain: CD28-based (axi-cel and brexu-cel) versus 4-1BB-based (tisa-cel and liso-cel) therapies excluding the three investigational CAR T cells. Statistical tests were done at the two-tailed α level of 0.05. Data were analyzed using R software package, release 4.3.3 (R Foundation for Statistical Computing, Vienna, Austria).

## Results

### Patient characteristics at infusion

We retrospectively analyzed data from 539 patients infused with CD19 directed CAR T cell between September 2017 and August 2023 with a median follow-up of 25 months (95% CI, 24–29). Patients’ characteristics before lymphodepletion (preLD) are shown in Table 1. The median age was 63 years old (range 17–87). Patients were mostly treated for DLBCL (*n *= 284, 53%), transformed indolent B-cell lymphomas (*n *= 98, 18%), indolent lymphoma (*n *= 66, 12,2%), or mantle cell lymphoma (*n *= 30, 5.6%). They received either axi-cel (*n *= 319, 59,2%), tisa-cel (*n *= 144, 26,6%), liso-cel (*n *= 43, 8%), brexu-cel (*n *= 30, 5.6%), or an investigational CD19 directed CAR T cells (*n *= 3, 0.6%). The median number of previous lines of therapy was 2 (range 1–11), median preLD LDH was 254U/I (IQR 206–328), median preLD MCV was 94 (IQR 89–98), median preLD CAR-HEMATOTOX score was 2 (range: 0–6). A total of 467 (86.6%) patients experienced CRS (≥grade 3: 6.5%), and 191 (35.4%) patients developed ICANS (≥grade 3: 8.9%). In the whole cohort, 29 patients developed a t-MN including 11 t-AML (38%) and 18 t-MDS (62%).

**Table 1:** Study population characteristics.

Total	*N *= 539
Patients	
Age – years, median (range)	63 (17–87)
Sex – Male, *n* (%)	335 (62.1)
Treatment center, *n* (%)	
Lyon & St Etienne	327 (60.7)
Lille	183 (34.0)
Caen	29 (5.4)
Histology, *n* (%)	
DLBCL NOS	284 (52.7)
GC	37 (6.9)
ABC	38 (7.1)
Not specified	209 (38.8)
Transformed indolent B-cell lymphoma	98 (18.1)
Mantle cell lymphoma	30 (5.6)
Follicular/marginal zone lymphoma	66 (12.3)
Primary mediastinal B-cell lymphoma	20 (3.7)
Richter transformation	18 (3.3)
Others	23 (4.3)
CAR T cell used, *n* (%)	
Axi-cel	319 (59.2)
Brexu-cel	30 (5.6)
Tisa-cel	144 (26.6)
Liso-cel	43 (8.0)
Other/experimental products	3 (0.6)
Data prior to lymphodepletion	
LDH (U/L) – median (IQR)	256 (206–328)
MCV (fl) – median (IQR)	94 (89–98)
Hemoglobin (g/dL) – median (IQR)	10.7 (9.25–12.1)
ANC (G/L) – median (IQR)	2.10 (1.10–3.8)
Platelet (G/L) – median (IQR)	120 (52.0–200)
ALC (G/L) – median (IQR)	0.700 (0.47–1.05)
CRP (mg/L) – median (IQR)	6.00 (2.00–23.0)
Ferritin (μg/L) – median (IQR)	343 (155–827)
CAR HEMATOTOX Score – median (IQR)	2 (2–3)
Median prior lines of therapy (IQR)	2 (2–3)
Prior autologous HCT, *n* (%)	95 (17.6)
Prior allogenic HCT, *n* (%)	4 (0.7)
Toxicity, *n* (%)	
Any grade CRS *n* (%)	467 (86.6)
Severe CRS *n* (%)	35 (6.5)
Any grade ICANS *n* (%)	191 (35.4)
Severe ICANS *n* (%)	48 (8.9)

*ABC* Activated B-Cell, *ALC* Absolute Lymphocyte Count, *ANC* Absolute Neutrophil Count, *axi-cel* Axicabtagene Ciloleucel, *brexu-cel* Brexucabtagene Autoleucel, *CAR T* cell product (CD28 vs 41BB), *CRP* C-Reactive Protein, *CRS* Cytokine Release Syndrome, *DLBCL* Diffuse Large B-Cell Lymphoma, *FL* Follicular Lymphoma, *GC* Germinal Center, *HCT* Hematopoietic Cell Transplant, *ICANS* Immune Effector Cell-Associated Neurotoxicity Syndrome, *liso-cel* Lisocabtagene Maraleucel, *MCL* Mantle Cell Lymphoma, *MCV* Mean Corpuscular Volume, *MZL* Marginal Zone Lymphoma, *NOS* Not Otherwise Specified, *PMBL* Primary Mediastinal B-Cell Lymphoma, *tDLBCL* Transformed Diffuse Large B-Cell Lymphoma, *tisa-cel* Tisagenlecleucel, *t-MN* Therapy-Related Myeloid Neoplasm.

### Outcome after CD19 CAR T cells infusion

**Fig. 1:**
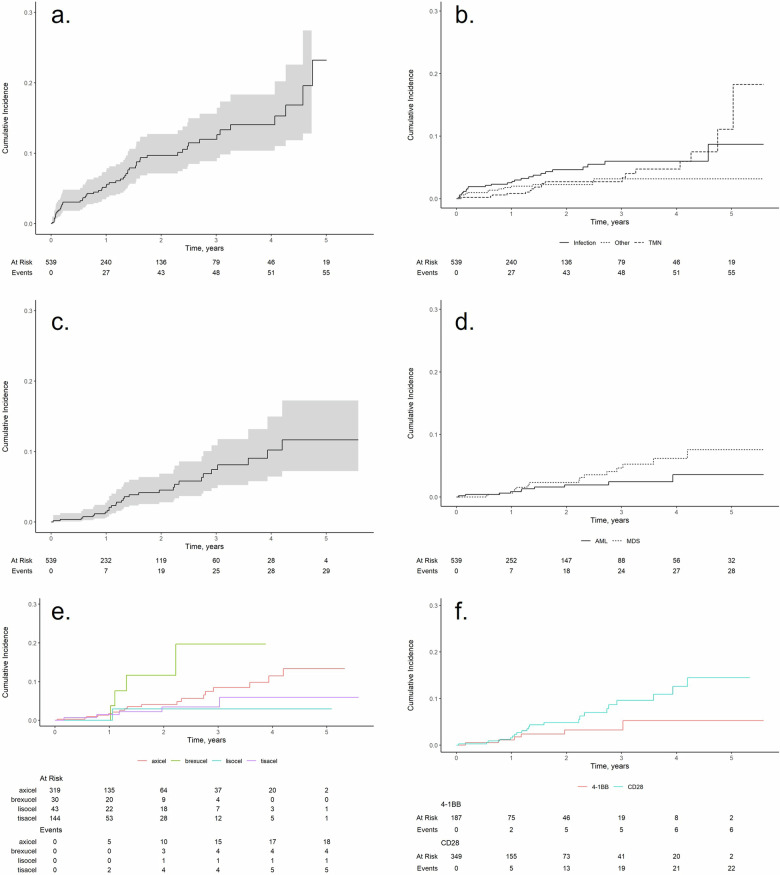
Non-relapse mortality (NRM) and therapy-related myeloid neoplasms (t-MN) incidences curves. **a** Incidence of NRM. **b** Incidence of NRM caused by infection, t-MN or other causes. **c** Incidence of t-MN with death and relapse treat as competing risk. **d** Incidence of t-AML and t-MDS with death and relapse treat as competing risk. **e** Incidence of t-MN depending of the CAR T cell used. **f** Incidence of t-MN depending of the co-stimulatory domain of the CAR T cells (4-1BB or CD28).

In the whole cohort, the overall response rate at 1 month was 79% with 61% of complete response CR (response rate according to histology is shown on Supplementary Fig. 1S). The median OS and PFS were 46.1 (95% CI, 32.3–57) and 11.8 months (95% CI, 7.8–18.4), respectively (Supplementary Fig. 2S). Cumulative incidences of NRM at 1, 2, 3 and 4 years was 5.4% (95% CI, 3.6–7.6), 9.7% (95% CI, 7.1–13), 12% (95% CI, 8.9–16) and 14.0% (95% CI, 10–18.0), respectively (Fig. 1a). At time of data cut-off, a total of 57 patients had died of NRM, mostly due to infection (*n *= 22, 39%), then t-MN (*n *= 19, 33%) (Fig. 1b).

Cumulative incidences of t-MN at 1, 2, 3 and 4 years in responder patients, was 1.4% (95% CI, 0.63–2.8), 4.5% (95% CI, 2.8–6.9), 7.5% (95% CI, 4.8–11) and 10% (95% CI, 6.4–15) respectively (Fig. 1c). Pre-lymphodepletion characteristics of patients based on t-MN status are shown in Supplementary Table 2S. When stratified by t-MN type, the median time to diagnosis was 14.2 months (IQR: 10–21.9 months) for t-AML and 15.8 months (IQR: 12.6–21.9 months) for t-MDS (Fig. 1d). The cumulative incidence of t-MN 2 years after treatment with liso-cel, tisa-cel, axi-cel, or brexu-cel was 3.0% (95% CI, 0.21–13), 3.5% (95% CI, 1.1–8.2), 4.1% (95% CI, 2.1–7.2), and 12% (95% CI, 2.8–27), respectively (Fig. 1e). When categorized by co-stimulatory domain, the cumulative incidence of t-MN for 4-1BB CAR T cells (tisa-cel and liso-cel) was 1.1% (95% CI, 0.22–3.7) at 1 year, 3.2% (95% CI, 1.2–7.1) at both 2 and 3 years, and 5.2% (95% CI, 1.2–12) at 4 years. For CD28 CAR T cells (axi-cel and brexu-cel), the cumulative incidence was 1,6% (95% CI, 0.6–3.5) at 1 year, 4.8% (95% CI, 2.7–7.9) at 2 years, 9.6% (95% CI, 2.7–7.9) at 3 years, and 13% (95% CI, 7.4–19) at 4 years (Fig. 1f).

### Univariate analyses of factors associated with the occurrence of t-MN

In univariate analyses, higher age at reinfusion was significantly associated with the higher occurrence of t-MN (HR 1.04; 95% CI 1.01–1.07, *p *< 0.01), as was the higher preLD MCV (HR 1.10; 95% CI 1.05–1.16, *p *< 0.01) and higher ICANS grade (HR 1.32; 95% CI 1.01–1.74, *p *= 0.04) (Table 2).

**Table 2:** Univariate cause-specific Cox model for t-MN.

Variable	HR	95% CI1	*p*-value
Age	1.04	1.01–1.07	**<0.01**
CAR-HEMATOTOX score	1.14	0.78–1.22	0.8
MCV	1.10	1.05–1.16	**<0.01**
LDH	1.10	0.70–1.72	0.7
ALC	1.10	0.7–1.72	0.7
Number of prior lines	1.11	0.98–1.25	0.11
CAR T cell product (CD28 vs 41BB)	1.82	0.75–4.34	0.2
CRS grade	0.88	0.64–1.2	0.4
ICANS grade	1.32	1.01–1.74	**0.04**

Bold values represent statistical significance *p* < 0.05.

No statistical association was found between the occurrence of t-MN and CAR T cell products, CAR-HEMATOTOX score, CRS grade, preLD LDH or ALC. The number of prior lines showed a trend toward an increased occurrence of t-MN (HR 1.11; 95% CI 0.98–1.25, *p *= 0.11), though this did not reach statistical significance at the 0.05 level.

### Factors associated with occurrence of t-MN after propensity score matching

Since age and prior chemotherapy have already been described as independent factors for developing t-AML and t-MDS, the cohort was matched based on age and the number of prior therapy lines before CAR T cell treatment.

In the matched population, higher preLD MCV (HR 1.10; 95% CI 1.03–1.16, *p *< 0.01) and higher ICANS grade (HR 1.26; 95% CI 1.06–1.50, *p *< 0.01), were still significantly associated with the occurrence of t-MN. Additionally, the presence of any grade ICANS was also associated with a higher occurrence of t-MN (HR 2.52; 95% CI 1.23–5.09, *p *= 0.01). A trend toward a higher occurrence of t-MN was observed with CD28 co-stimulatory domain CAR T cells (axi-cel and brexu-cel; HR 2.15; 95% CI 0.89–5.22, *p *= 0.09), though the association was not statistically significant (Table 3).

**Table 3:** Univariate cause-specific Cox model for t-MN after matching on age and number of prior lines before CAR T cells therapy.

Variable	HR	95% CI	*p*-value
CAR HEMATOTOX score	1.2	0.96–1.51	0.11
MCV	1.10	1.03–1.16	**<0.01**
LDH	1.1	0.70–1.72	0.7
ALC	0.84	0.39–1.81	0,7
CAR T cell product (CD28 vs 41BB)	2.15	0.89–5.22	0.09
CRS grade	0.94	0.62–1.42	0.8
ICANS grade	1.26	1.06–1.50	**<0.01**

Bold values represent statistical significance *p* < 0.05.

### t-MN characteristics and treatment

**Fig. 2:**
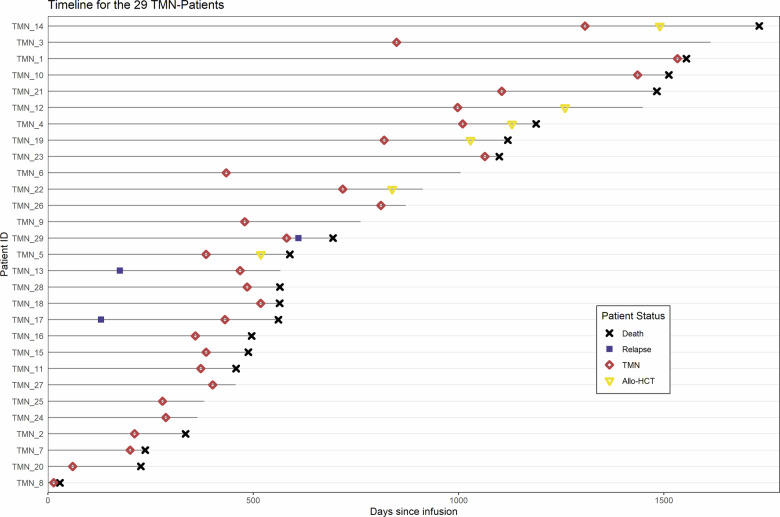
Individual courses of therapy-related myeloid neoplasms (8-MN) patients from CAR T cells infusion.

A total of 29 patients were diagnosed with t-MN, 11 patients with t-AML (38%) and 18 with t-MDS (62%). At t-MN diagnosis, the median age was 68 (range 53–80). The median time between CAR T cell infusion and t-MN diagnosis was 15.6 months (range 1.0–56.9). At t-MN diagnosis, median hemoglobin level was 9.1 g/dL (IQR 7.8–9.9), MCV was 96fl (IQR 83–111), absolute neutrophil count was 0.9 G/L (IQR 0.47–1.52) and platelet count was 47 G/L (IQR 28–59). Clinical courses of individual patients who developed t-MN from CAR T cells infusion are shown in Fig. 2.

Among the 11 t-AML cases, 91% (*n *= 10) were classified as intermediate (*n *= 3) or adverse (*n *= 7) risk according to ELN 2022 classification, and one patient showed an inv [16] on karyotype. Morphologically, 46% (*n *= 5) of patients had marrow infiltration with ≥60% blasts, as assessed by bone marrow aspiration. Immunophenotypic data were available for 73% (*n *= 8) of patients, showing predominantly myelomonocytic differentiation in 50% (*n *= 4). Cytogenetic analysis revealed abnormal karyotypes in 100% (*n *= 11) of patients, with 64% (*n *= 7) classified as complex karyotypes. Monosomies were observed in 36% (*n* = 4) of cases, most commonly involving chromosomes 5 and 7. Specific chromosomal rearrangements were found including inv [16] (*n *= 1) and KMT2 rearrangement (*n *= 1). NGS results were available for 82% (*n *= 9) of patients. Among these, *TP53* mutations were detected in 33% (*n *= 3), *TET2* mutations in 36% (*n *= 4), and *DNMT3A* mutations in 18% (*n *= 2). Of the 11 t-AML cases, 4 received intensive chemotherapy, 4 were treated with azacitidine and venetoclax, 2 received azacitidine alone, and for one patient, supportive care was chosen. Two of these patients underwent allogeneic stem cell transplantation, and only 3 patients are still alive.

Among the 18 t-MDS cases, 11 (61%) had high or very high R-IPSS scores. Cytogenetic analysis revealed that 67% (*n *= 12) of cases harbored monosomal or complex karyotypes, with frequent anomalies involving chromosomes 5 (*n *= 6) and 7 (*n *= 7). Among the 15 cases with available sequencing, *TP53* mutations were detected in 80% (*n *= 12). Additional recurrent mutations included *DNMT3A* (27%, *n *= 4), *PPM1D* (27%, *n *= 4), *ASXL1* (20%, *n *= 3), and *SF3B1* (13%, *n *= 2). Of the 18 t-MDS cases, 6 (33%) underwent allogeneic stem cell transplantation, 4 (22%) received azacitidine based chemotherapy and 5 (28%) received support care only.

**Fig. 3:**
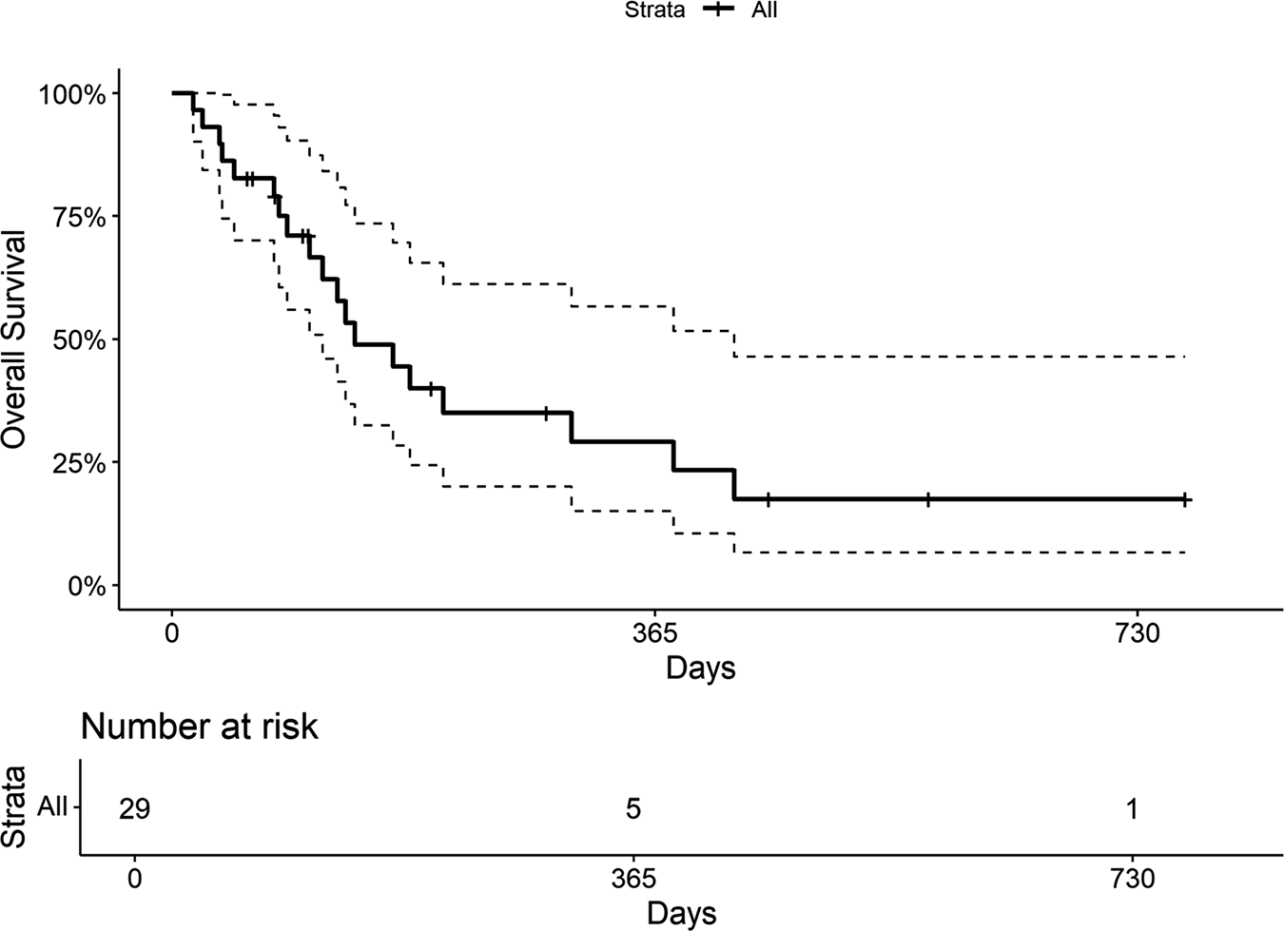
Overall survival of the t-MN cohort, from t-MN diagnosis.

In the full cohort of t-MN patients (t-AML and t-MDS), median OS from t-MN diagnosis was 4.5 months (95% CI, 3.7–14.2), with a 1-year survival rate of 29.2% (95% CI, 15.0–56.6) (Fig. 3). The median OS from t-MN diagnosis was 4.5 months for patients with t-AML and 6.7 months for those with t-MDS.

### Clonal evolution of myeloid subclones during CAR T cells treatment

We retrospectively analyzed pre-CAR T cell NGS data from various biological materials, as outlined in our methods. Among the 29 patients who developed t-MN, 14 patients (48.2%) had NGS data available prior to CAR T cell infusion (9 t-MDS and 5 t-AML). Of these, 12 patients (85.7%) exhibited pre-existing mutations (8 t-MDS and 4 t-AML). The most frequently identified mutations included *TP53* (50%), *DNMT3A* (50%), and *PPM1D* (33%).

**Fig. 4:**
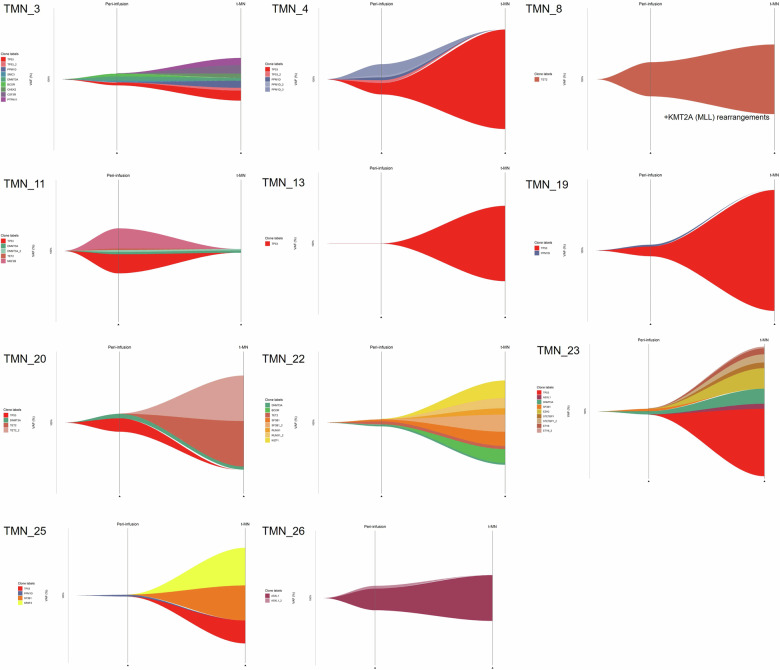
Clonal evolution before and after CAR T cell infusion.

NGS results at t-MN diagnosis were available for all 14 patients with pre-treatment NGS data. Among these, 10 patients (71.4%) retained at least one mutation identified prior to CAR T cell therapy. Patients with pre-treatment *TP53* mutations retained the *TP53* clone post-transformation in most cases (4 out of 6, 66%). The pre- and post-CAR T cell infusion VAFs are shown in Supplementary Fig. 4S. Two patients with a *TP53* mutation pre-treatment did not show this mutation at t-MN onset (Fig. 4). The only patient who developed AML without pre-existing mutations presented with favorable-risk AML. The other three patients, without pre-treatment mutations (*n* = 1) or with different post-treatment mutations (*n* = 2), developed t-MN with *TP53* mutations.

The median VAF percentage for *TP53* mutation before and after CAR T cell therapy were 4,4% and 38,5% respectively. Patients with pre-existing *TP53* mutations did not develop t-MN significantly faster, with a median time to onset of 12.7 months (IQR: 6.9–26.9), compared to 19.1 months (IQR: 13–27) for patients without *TP53* mutations (*p *= 0.72) (Supplementary Fig. 4S).

## Discussion

Our study is the first multicentric analysis to identify risk factors for the development of therapy-related myeloid neoplasms after CD19 directed CAR T cell therapy and to apply a competing risk approach for incidence estimation. CAR T cells are a revolution in hematology and are increasingly being used sooner and for more patients. Many studies have focused on early toxicities such as CRS, ICANS, infection or cytopenias. The four FDA approved CD19 directed CAR T cells, axi-cel, tisa-cel, liso-cel and brexu-cel have now had several years of follow-up, showing a persistent response in many patients. Given these long-responders the question of later toxicity arises. In clinical trials, the appearance of new hematological diseases is often described as unrelated to CAR T cell treatment. But recently, the FDA has warned of secondary primary malignancies occurring in these patients [30]. We focused our work on myeloid malignancies such as AML and MDS, as various reports align that these are the most common secondary cancers following CAR T cell therapy [22, 31].

In our cohort, after a median follow up of 2 years, we described a cumulative incidence of t-MN at 2 years of 4.5% and the projected 4-year incidence was 10% after treating relapse or death as competing risk. Considering incidence and relapses as competing risks allows us to focus on patients who responded to treatment and potentially cured of their lymphoma, avoiding an artificially reduced incidence rate. In comparison, Metayer et al. described a cumulative incidence of 3,9% at 7 years in a cohort of 1784 patient who underwent transplantation for NHL [32]. An other study showed a cumulative incidence of MDS/AML of 6.6% and 9.5% at 5 and 8 years respectively after chemotherapy in a cohort of indolent NHL [33]. These t-MN appear to have cytogenetic and molecular characteristics associated with a poorer prognosis compared to t-MNs typically described in the literature following chemotherapy. For instance, Belhabri et al. reported that 42% of AML cases post-chemotherapy harbored complex karyotypes, whereas 64% of cases in our cohort showed similar features. Additionally, the median survival for AML in their study was 9 months compared to 4.5 months in our cohort [34]. Also, the time of onset appears different, occurring much sooner after CAR T cell therapy compared to after chemotherapy [35].

In our study, the univariate analyses suggested an association between age and the occurrence of t-MN. This association has already been described for t-MN after CAR T cells therapy and after chemotherapy [33, 36]. Knowing that age and number of prior lines of therapy are usually described as risk factors of t-MN, we used a propensity score for matching analysis [37]. A higher preLD MCV and ICANS grade were significantly associated with higher occurrence of t-MN, before and after matching analysis. The MCV is known as being predictive of risk for myeloid malignancy in clonal hematopoiesis and recently was described as associated with t-MN after CAR T cells therapy [38, 39]. The explanation why ICANS grade is associated with t-MN remains unclear. The presence of clonal hematopoiesis before CAR T cells is not commonly associated with higher ICANS grade [40]. Moreover, the experience with the SARS-CoV-2 virus, among others, has demonstrated a close link between inflammation and the development of clonal hematopoiesis [41, 42]. One hypothesis is that in patients with high-risk CHIPs —typically older patients with an increased MCV— a high ICANS grade, correlated with elevated cytokine levels, may enable clonal selection and blast proliferation as it has been described with a CD123 directed CAR T cells [43]. Although this association did not reach statistical significance, we observed that CAR T cells with a CD28 co-stimulatory domain (axi-cel and brexu-cel) were associated to a higher risk of developing t-MN. Given that these products are known to cause higher rates and more severe neurotoxicity, the ICANS grade may simply reflect the use of CAR T cells with a CD28 co-stimulatory domain and the sample size in this study may have been insufficient to detect a significant risk for axi-cel and brexu-cel. CRS, which is common but rarely severe, was not associated with an increased risk of t-MN [44, 45].

To determine whether clonal selection was the primary mechanism driving the development of t-MN, we examined mutations in patients with t-MDS and t-AML before and after CAR T cell therapy. Among the 14 patients with NGS available prior to CAR T cell infusion, 12 (85.7%) exhibited pre-existing mutations, with *TP53* being the most frequent and almost always retained at the time of t-MN diagnosis. This underscores the pivotal role of *TP53* in clonal persistence and progression. Of the patients with pre-existing mutations, most maintained these mutations after CAR T cell therapy. For the 4 patients who did not, one presented with favorable-risk AML, while three developed *TP53*-mutated t-MN. This raises the possibility that *TP53* clones may have been present at very low levels pre-treatment but were undetected, as suggested by other studies on *TP53* clonal evolution under selective pressures, such as lenalidomide treatment [46].

A recent meta-analysis showed an NRM rate attributable to infections of 50.9%, followed by 7.8% from new malignancies, one-third of which were secondary to MDS or AML [22]. These findings align with a French cohort that focused on NRM [31]. In our cohort, we considered fatal infectious events occurring after the onset of a t-MN as t-MN-related deaths explaining why the rate of NRM due to infections was lower (37%), while NRM due to t-MN was higher (33%).

This study is limited by its retrospective nature, which may introduce inherent biases in data collection and interpretation. The rarity of t-MN events constrained the statistical power needed for multivariate analysis, limiting our ability to evaluate multiple risk factors. The median follow-up of this study is 2 years is relatively short for comprehensively assessing late toxicities. Variations in diagnostic practices and genetic testing across the centers may also have affected the consistency of cytogenetic and molecular findings. Also, as this cohort includes patients treated with investigational CAR T-cell products and across different treatment eras, heterogeneity in treatment protocols and supportive care may have influenced outcomes. Finally, while propensity score matching was applied to mitigate confounding, residual biases cannot be entirely excluded.

In conclusion, there is growing interest in understanding the long-term complications following CAR T cell therapy. While prior studies have investigated secondary primary malignancies, our study is the first to specifically detail and identify risk factors for secondary myeloid neoplasms after CD19 directed CAR T cell for B-NHL. After using competitive risk for relapse and death, the cumulative incidence of t-MN was 4.5% at 2 years and 10% at 4 years and we also demonstrated that, after matching for age and the number of prior therapy lines, preLD MCV and ICANS grade were associated with an increased risk of t-MN. Further research is needed to validate these findings and to explore the precise impact of the co-stimulatory domain and cytokines on t-MN development.

## Supplementary information

Supplemental Materials

## Data Availability

The data that support the findings of this study are available on request from the corresponding author.
