# Supplementary material for: Myeloid neoplasms after CD19-directed CAR T cells therapy in long-term B-cell lymphoma responders, a rising risk over time?
Source: Leukemia. 2025 Apr 24;39(7):1714–22. doi: 10.1038/s41375-025-02605-7 (PMC12208870; doi:10.1038/s41375-025-02605-7)

**Supplementary data**

**Myeloid neoplasms after CD19-directed CAR T cells therapy in long-term B-cell lymphoma responders, a rising risk over time?**

Nicolas Gazeau et al.

**Table 1S: Next-generation sequencing panel used in Lyon and Lille.**

| **Targeted next-generation sequencing panel developed/performed in Lyon** | **Targeted next-generation sequencing panel developed/performed in Lille** |
| --- | --- |
| ABL1(NM_005157.5),ANKRD26(NM_014915.3),ARID1A(NM_006015.5),ASXL1(NM_015338.5),ASXL2(NM_018263.4),ATM(NM_000051.3),B2M(NM_004048.2),BCL2(NM_000633.2),BCL6(NM_001130845.1),BCOR(NM_001123385.1),BCORL1(NM_001184772.2),BIRC3(NM_182962.2),BRAF(NM_004333.4),BTK(NM_000061.2),CALR(NM_004343.3),CARD11(NM_001324281.1),CBL(NM_005188.2),CCND1(NM_053056.2),CCND3(NM_001760.4),CD58(NM_001779.2),CD79A(NM_001783.3),CD79B(NM_001039933.2),CDKN2A(NM_058195.2),CDKN2B(NM_004936.3),CEBPA(NM_004364.4),CIITA(NM_001286402.1),CRBN(NM_016302.2),CREBBP(NM_004380.2),CSF3R(NM_156039),CXCR4(NM_001348056.1),DDX41(NM_016222.4),DNMT3A(NM_022552.4),EP300(NM_001429.3),ETNK1(NM_018638.4),ETV6(NM_001987.4),EZH2(NM_004456.4),FBXW7(NM_033632.3),FLT3(NM_004119),FOXO1(NM_002015.3),GATA2(NM_032638),GNA13(NM_006572.5),GNB1(NM_002074.5),HRAS(NM_005343.2),ID3(NM_002167.4),IDH1(NM_005896),IDH2(NM_002168),IKZF1(NM_006060.5),IRF4(NM_002460.3),JAK2(NM_004972.3),JAK3(NM_000215.4),KIT(NM_000222),KLF2(NM_016270.3),KMT2A(NM_001197104.1),KMT2D(NM_003482.3),KRAS(NM_033360),MAP2K1(NM_002755.3),MECOM(NM_004991.3),MEF2B(NM_001145785.1),MPL(NM_005373.2),MYC(NM_002467.4),MYD88(NM_002468.4),NFE2(NM_006163.2),NFKBIE(NM_004556.2),NOTCH1(NM_017617.3),NOTCH2(NM_024408.3),NPM1(NM_002520),NRAS(NM_002524),PAX5(NM_016734.2),PDGFRB(NM_002609.3),PHF6(NM_001015877.1),PIM1(NM_001243186.1),PLCG2(NM_002661.4),PPM1D(NM_003620.4),PRDM1(NM_001198.3),PTEN(NM_000314.6),PTPN11(NM_002834.3),RAD21(NM_006265.2),RHOA(NM_001313941.1),RRAGC(NM_022157.3),RUNX1(NM_001754.4),SETBP1(NM_015559),SETD2(NM_014159.6),SF3B1(NM_012433.2),SH2B3/LNK(NM_005475.2),SMC1A(NM_006306.3),SMC3(NM_005445.3),SOCS1(NM_003745.1),SRSF2(NM_003016),STAG1(NM_005862.2),STAG2(NM_001042749.2),STAT3(NM_139276.2),STAT5B(e8,e16,e17)(NM_012448.3),STAT6(NM_001178078.1),SUZ12(NM_015355.3),SYK(NM_001174167.2),TCF3(NM_003200.4),TET2(NM_001127208),TNFAIP3(NM_001270507.1),TNFRSF14(NM_003820.3),TP53(NM_001126112),TRAF2(NM_021138.3),TRAF3(NM_145725.2),U2AF1(NM_006758),UBA1(NM_003334.3),UBTF(NM_014233.4)WT1(NM_024426),XBP1(NM_001079539.1),XPO1(NM_003400.3),ZRSR2(NM_005089.3) | *ABL1*(NM_005157.4),*ANKRD26*(NM_0149), *ASXL1*(NM_015338.5),*ASXL2*,(NM_018263.4),*ATRX*(NM_000489.4),*BCOR*(NM_001123385.1),*BCORL1*(NM_021946.4),*BRAF*(NM_004333.4),*CALR*(NM_004343.3),*CBL*(NM_005188.3),*CEBPA*(NM_004364.4),*CHEK2*(NM_001005735.1),*CREBBP*(NM_004380.2),*CRLF2*(NM_022148.3),*CSF3R*(NM_156039.3),*CUX1*(NM_181552.3), *DDX41*(NM_016222.3),*DHX15*(NM_001358.2),*DHX34*(NM_014681.5),*DNMT3A*(NM_022552.4),*EP300*(NM_001429.3),*ETNK1*(NM_018638.4),*ETV6*(NM_001987.4),*EZH2*(NM_004456.4),*FBXW7*(NM_033632.3),*FGFR1*(NM_001174067.1),*FLT3*(NM_004119.2),*GATA1*(NM_002049.3),*GATA2*(NM_032638.4),*GNAS*(NM_000516.5),*GNB1*(NM_002074.4),*HRAS*(NM_005343.3),*IDH1*(NM_005896.3),*IDH2*(NM_002168.3),*IKZF1*(NM_006060.5),*IL7R*(NM_002185.3),*JAK1*(NM_002227.3),*JAK2*(NM_004972.3),*JAK3*(NM_000215.3),*KDM6A*(NM_001291415.1),*KIT*(NM_000222.2),*KMT2A*(NM_001197104.1),*KRAS*(NM_033360.3),*LUC7L2*(NM_001244585.1),*MBD4*(NM_003925.2),*MPL*(NM_005373.2),*MYC*(NM_002467.4),*NF1*(NM_001042492.2),*NFE2*(NM_001136023.2),*NIPBL*(NM_133433.3),*NOTCH1*(NM_017617.4),*NPM1*(NM_002520.6),*NRAS*(NM_002524.4),*PAX5*(NM_016734.2),*PDGFRA*(NM_006206.5),*PDGFRB*(NM_002609.3),*PHF6*(NM_001015877.1), *PIGA*(NM_002641.3),*PPM1D*(NM_003620.3),*PRPF8*(NM_006445.3),*PTEN*(NM_000314.6),*PTPN11*(NM_002834.4),*RAD21*(NM_006265.2),*RIT1*(NM_006912.5),*RUNX1*(NM_001754.4),*SAMD9*(NM_017654.3), *SAMD9L*(NM_152703.3),*SETBP1*(NM_015559.2),*SETD2*(NM_014159.6),*SF3B1*(NM_012433.3),*SH2B3*(NM_005475.2),*SMC1A*(NM_006306.3),*SMC3*(NM_005445.3),*SRP72*(NM_006947.3),*SRSF2*(NM_003016.),*STAG2*(NM_001042749.2),*STAT3*(NM_003150.3),*STAT5A*(NM_003152.3),*STAT5B*(NM_012448.3),*TERC*NR_001566.1),*TERT*(NM_198253.2),*TET2*(NM_001127208.2),*TP53*(NM_001126112.2),*TYK2*(NM_003331.4),*U2AF1*(NM_006758.2),*UBA1*(NM_003334.3),*UBTF*(NM_014233,3*WT1*(NM_024426.4),*ZBTB7A*(NM_015898.3),*ZRSR2*(NM_005089.3) |

**Table 2S: Pre-lymphodepletion characteristics of patients with and without therapy-related myeloid neoplasms.**

|  | **No TMN** **(N=510)** | **TMN** **(N=29)** |
| --- | --- | --- |
| **Patients** | | |
| Age – median (range) | 63.0 [53.0, 70.0] | 66.0 [57.0, 71.0] |
| Sex – Male, n (%) | 317 (62.2%) | 18 (62.1%) |
| **CAR-T cell used, n (%)** | | |
| Axicel | 301 (59.0%) | 18 (62.1%) |
| Brexucel | 26 (5.1%) | 4 (13.8%) |
| Tisacel | 139 (27.3%) | 5 (17.2%) |
| Lisocel | 42 (8.2%) | 1 (3.4%) |
| Other / experimental products | 2 (0.4%) | 1 (3.4%) |
| **Data Prior to Lymphodepletion** | | |
| LDH (UI/L) – median (IQR) | 256 (206 **–** 330) | 260 (205 **–** 299) |
| MCV (fl) – median (IQR) | 94.0 (89 **–** 97) | 99.2 (93.5 **–** 102) |
| Hemoglobin (g/dL) – median (IQR) | 10.7 (9.3 **–** 12.1) | 10.5 (9.1 **–** 11.7) |
| ANC (G/L) – median (IQR) | 2.08 (1.06 **–** 3.80) | 2.59 (1.81 **–** 3.59) |
| Platelet (G/L) – median (IQR) | 119 (50.3 **–** 199) | 148 (87 **–** 216) |
| ALC (G/L) – median (IQR) | 0.7 (0.47 **–** 1.05) | 0.735 (0.4 **–** 0.998) |
| CRP (mg/L) – median (IQR) | 5.90 (2 **–** 23.6) | 6.1 (1.70, 14) |
| Ferritin (μg/L) – median (IQR) | 333 (151 **–** 798) | 584 (269 **–** 852) |
| CAR HEMATOTOX Score – median (IQR) | 2 (1 **–** 3) | 2 (1 **–** 3) |
| Median Prior Lines of Therapy | 2 (2 **–** 3) | 3 (2 **–** 3) |
| Prior Autologous HCT, n (%) | 84 (16.5%) | 11 (37.9%) |
| Prior Allogenic HCT, n (%) | 4 (0.8%) | 0 (0%) |

**Figure 1S: Response to CAR T cell therapy one month after infusion**

1. **For DLBCL and PMBL b) for indolent lymphoma (FL, MZL), c) for mantle cell lymphoma d) for transformed indolent lymphoma and Richter**


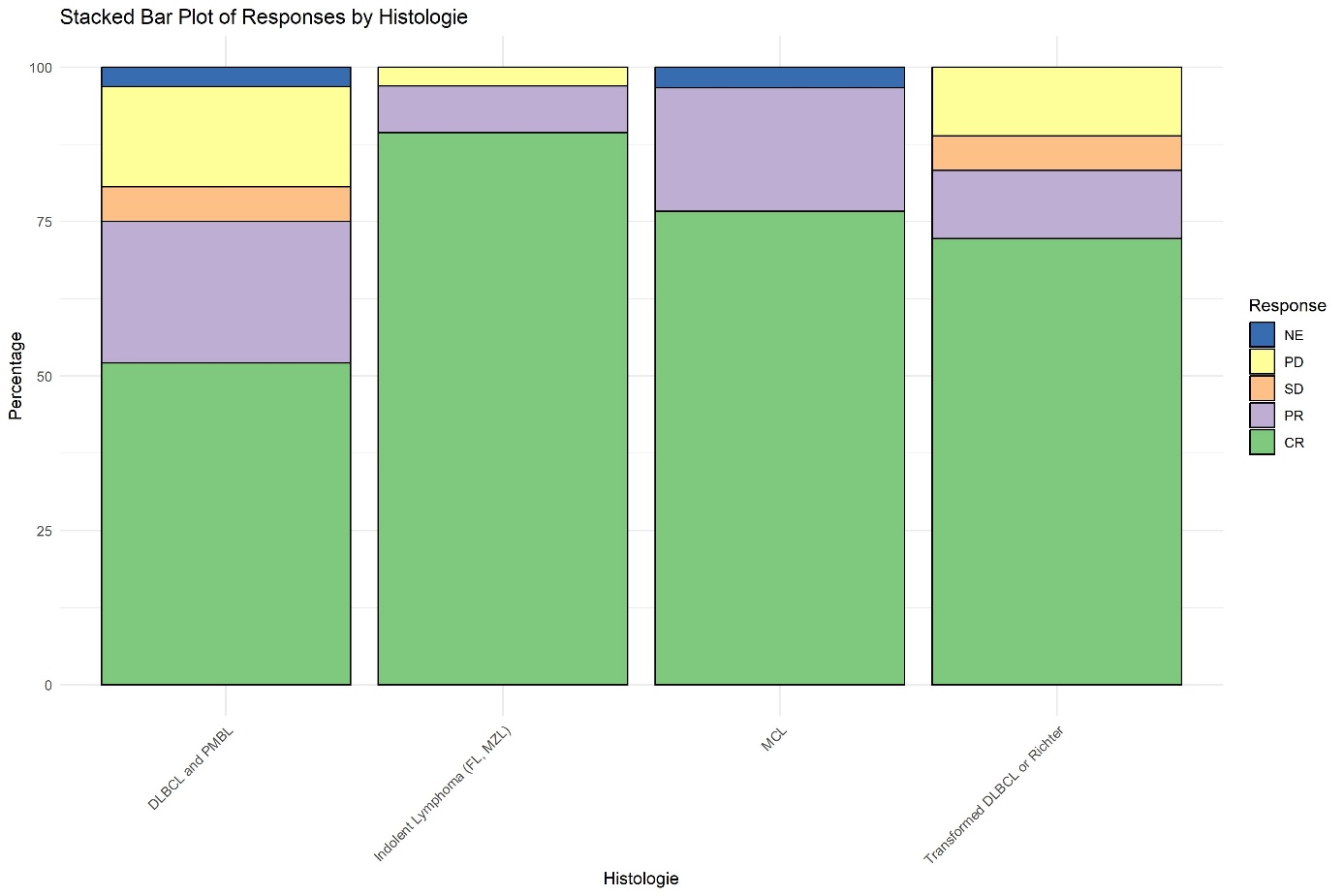


Abbreviations: CR, complete response, PR, partial response, SD, stable disease, PD , progressive disease, NE , not evaluable

**Figure 2S: PFS and OS of the entire cohort**

**
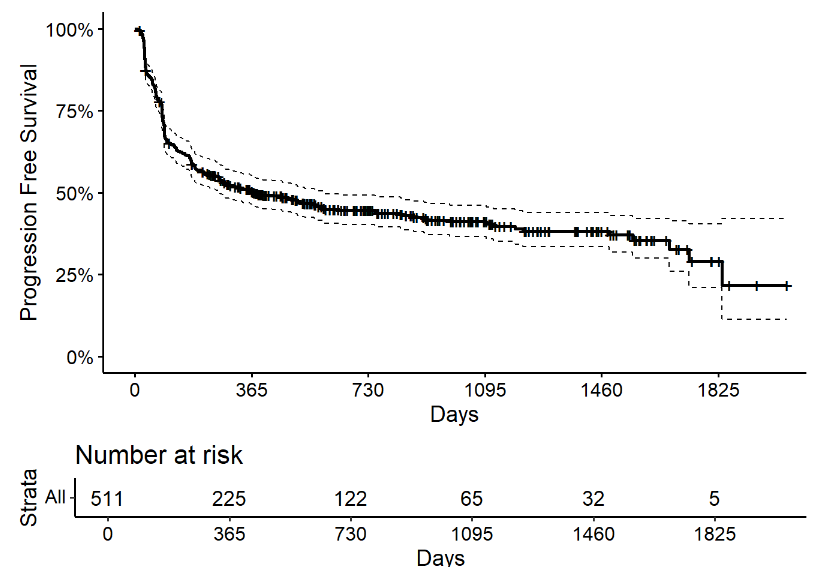

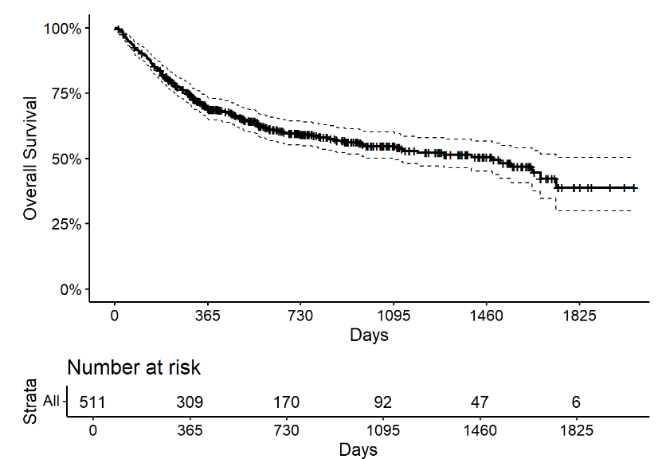
**

**Figure 3S: Box and whisker plot of variant allele frequencies of mutations pre- and post-CAR T-cell infusion in t-MN patients. Most frequent mutated genes were TP53, PPM1D and DNMT3A.**


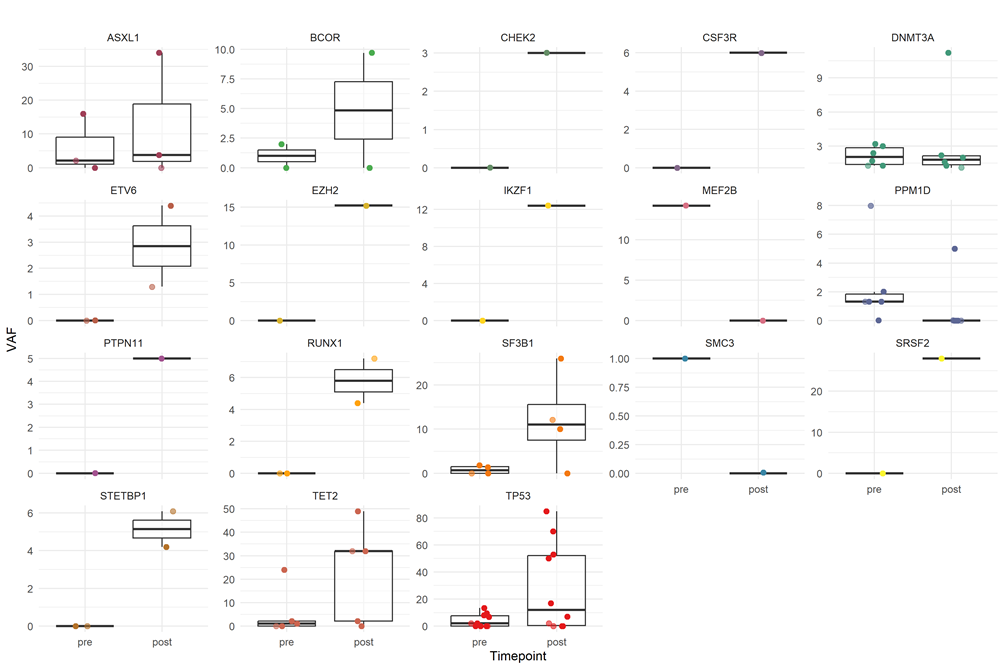


**Figure 4S: Cumulative incidence curve of t-MN incidence depending on TP53 Mutation Status**


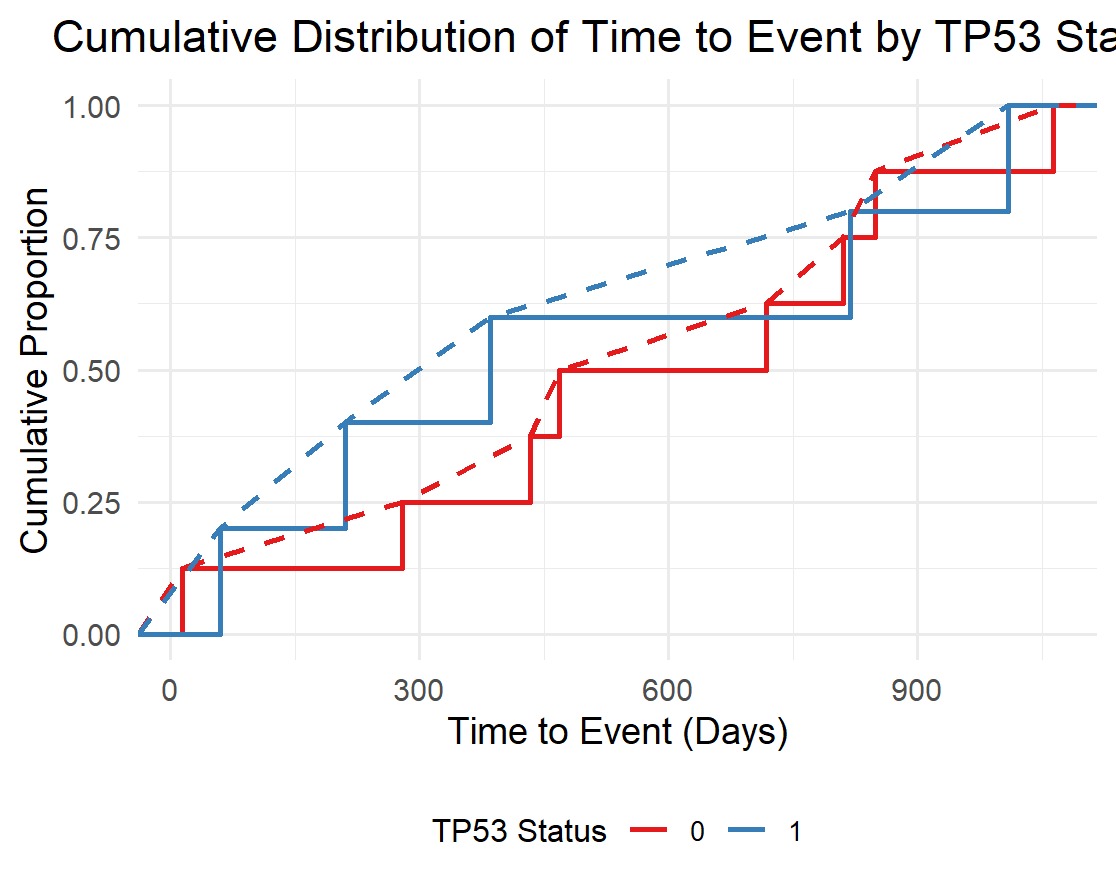

Supplement: Supplementary file 1 — Supplemental Materials [file 41375_2025_2605_MOESM1_ESM.docx]
